# Shifts in *Pseudomonas* species diversity influence adaptation of brown planthopper to changing climates and geographical locations

**DOI:** 10.1016/j.isci.2022.104550

**Published:** 2022-06-07

**Authors:** Ayushi Gupta, Deepak Kumar Sinha, Suresh Nair

**Affiliations:** 1 Plant-Insect Interaction Group, International Centre for Genetic Engineering and Biotechnology (ICGEB), Aruna Asaf Ali Marg, New Delhi 110067, India

**Keywords:** Entomology, Microbiology, plant biology

## Abstract

The brown planthopper (BPH) is a monophagous sap-sucking pest of rice that causes immense yield loss. The rapid build-up of pesticide resistance combined with the ability of BPH populations to quickly overcome host plant resistance has rendered conventional control strategies ineffective. One of the likely ways in which BPH adapts to novel environments is by undergoing rapid shifts in its microbiome composition. To elucidate the rapid adaptation to novel environments and the contributions of *Pseudomonas* toward insect survival, we performed *Pseudomonas*-specific *16S rRNA* gut-microbiome profiling of BPH populations. Results revealed the differential occurrence of *Pseudomonas* species in BPH populations with changing climates and geographical locations. Further, the observed variation in *Pseudomonas* species composition and abundance correlated with BPH survivability. Collectively, this study, while adding to our current understanding of symbiont-mediated insect adaptation, also demonstrated a complex interplay between insect physiology and microbiome dynamics, which likely confers BPH its rapid adaptive capacity.

## Highlights

• BPH, a major pest of rice, undergoes seasonal shifts in its microbiome composition
• *Pseudomonas* sp. in BPH microbiome varied with seasons and geographical locations
• *Pseudomonas* sp. composition and abundance correlated with BPH survivability
• Environment-guided microbial shifts drive rapid stress adaptations in BPH

## Graphical abstract

**Figure:**
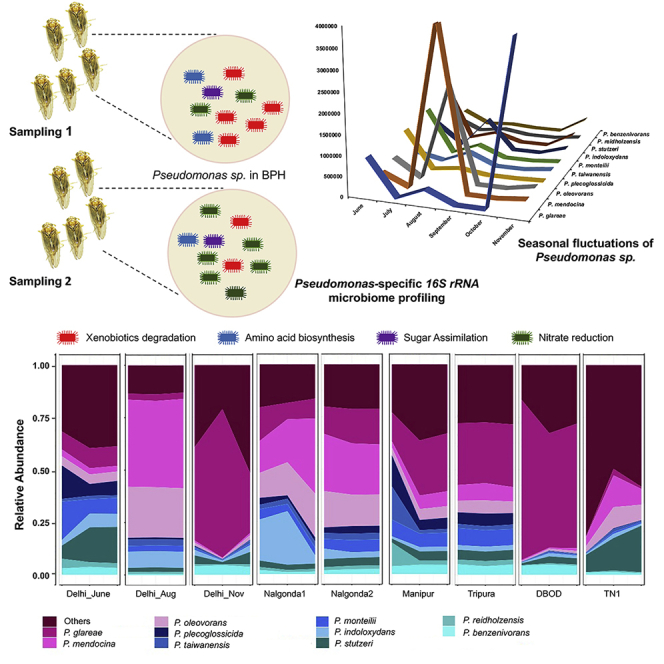

## Introduction

The intricate relationship of insects with their gut microbiome is largely implicated in the diversification and evolutionary success of various insect pests (Janson et al., 2008). The endosymbiotic bacteria that constitute the microbiome provide several nutritional benefits to their insect hosts by synthesizing various essential amino acids, metabolic compounds, and nutrients to compensate for the nutrient-poor diets many sap-sucking insects rely upon (Russell et al., 2014; Gupta and Nair, 2020). In addition, gut microbes are involved in the detoxification of xenobiotic compounds and in providing immunity and protection against various predators, pathogens, and parasites, and are, therefore, one of the primary determinants of insect’s fitness and longevity (Douglas, 2015; Wielkopolan and Obrêpalska-Stêplowska, 2016). Besides, the gut-associated bacteria also provide an additional flexible metabolic toolbox for facilitating the adaptation of insects to various insect-resistant plant varieties (Santos-Garcia et al., 2020). Results from several studies suggest that changes in the microbiome composition are linked to virulence adaptation in insects, as observed for aphids on wheat and leafhoppers and planthoppers on rice (Horgan et al., 2019). Collectively, these observations reveal the significance and worth of the contributions of gut microbes for insect survival.

Brown planthopper (*Nilaparvata lugens;* BPH), a monophagous sap-sucking insect, is a highly migratory pest capable of traversing long distances and has, therefore, invaded all the major rice-growing areas in the world. Over the past few years, it has become one of the major pests of rice, causing immense yield loss. Conventional strategies to control BPH are proving unsuccessful, and more importantly, populations of BPH are acquiring resistance to most pesticides and thereby rendering them ineffective within a few generations. Moreover, many rice varieties currently deployed in the field are largely susceptible to BPH due to the latter’s ability to rapidly overcome the host plant resistance. Therefore, it has become crucial to devise alternate methods and formulate a long-term pest control strategy for effectively managing the BPH outbreaks. In this regard, it is pertinent to investigate the mechanisms operating in the BPH, which endow it with the capacity to resist/tolerate pesticides and rapidly break down host plant resistance.

It is well established that the microbiome composition of insects is highly dynamic in terms of its structure, function, and composition and is likely influenced by several physical, physiological, and environmental factors (Gupta and Nair, 2020). Previous studies on the BPH microbiome, while indicating its dynamic nature, also revealed several bacterial and yeast-like endosymbionts that are associated with and vital for BPH survival (Tang et al., 2010). Additionally, it was observed that the bacterial species composition varies significantly between the BPH populations feeding on different rice varieties and between BPH biotypes (Tang et al., 2010). Similar observations have been made for aphids and whiteflies, where the community structure of secondary symbionts not only show remarkable variation between biotypes but these differences also contribute to their varied virulence and tolerance capacity to various biotic and abiotic stresses (Indiragandhi et al., 2010; Guyomar et al., 2018). Similarly, Santos-Garcia et al. (2020) have reported fluctuations in the “core” and “transient” bacterial communities dominated by the phylum Proteobacteria, Actinobacteria, and Firmicutes, when insects are reared on different hosts. They observed a significant increase in the relative abundance of *Mycobacterium* in whiteflies feeding on pepper plants. And since *Mycobacterium* is capable of degrading xenobiotics and secondary metabolites, therefore, an increase in its titer has been implicated in facilitating whitefly’s adaption to pepper. Similarly, several species of *Pseudomonas* have been reported to be associated with insecticide-resistant populations of *Spodoptera frugiperda* (de Almeida et al., 2017). Likewise, variation in *Pseudomonas* titers was reported between the lab- and field-collected populations of the Hessian fly (Bansal et al., 2014).

Collectively, these observations imply that the microbiome is actively involved in host switching and facilitating insect adaptation. Moreover, these studies indicate the implications of varied microbiome structure and composition across insect populations and between biotypes. Taking these observations into account, it is plausible to believe that the microbiome could likely contribute to the rapid adaptive response of BPH to various biotic and abiotic stresses. In addition, the fluctuations in the microbiome composition could also account for the varied virulence of BPH populations. This hypothesis is supported by the study by Malathi et al. (2018), where they reported that the insecticide-susceptible and -resistant BPH populations differ in their gut bacterial diversity. However, additional investigations are required to assess the variation in microbiome structure and composition across BPH populations as a response to the environment and their likely impact on its survival.

Several *Pseudomonas* species are known to facilitate insect survival on recalcitrant food sources. For instance, *Pseudomonas* species, i.e., *P. fulva* and *P.* *putida*, degrade caffeine (an alkaloid toxic to insects) in the gut of the coffee-berry borer, *Hypothenemus hampei* (Ceja-Navarro et al., 2015); *P. orientalis* exhibits lignin peroxidase activity in the wood-boring beetle, *Agrilus mali* (Bozorov et al., 2019), and thereby enabling its feeding on the host plant. Moreover, *Pseudomonas* sp*.* are also known to mediate some ecologically important traits of their insect hosts. Data from various studies have elucidated the host-protection role of *Pseudomonas*, e.g., *Pseudomonas sp*. present in the rove beetles produce the polyketide “pederin”, which not only reduces its palatability for predators but also prevents the entry of other pathogens and parasites (Kellner, 2003; Piel et al., 2004). Similar observations have been made in *Spodoptera litura*, where various siderophore producing *Pseudomonas* sp. showed antagonistic activity toward entomopathogenic fungi (Subhashini, 2015). Therefore, considering the varied roles undertaken by *Pseudomonas* sp. as a component of the insect microbiome, it is crucial to investigate the structure and composition of *Pseudomonas* species and their significance for the growth, development, and survival of BPH. Such studies could provide valuable information regarding the involvement of the microbiome in mediating the rapid adaptive stress responses in BPH.

Recent studies have indicated that the host plant and the source of the insect population drive the diversity of gut microbial communities in various insect pests such as *Spodoptera* and *Helicoverpa* (Jones et al., 2019)—both major insect pests of agricultural crops. And as *Pseudomonas* is a facultative symbiont which BPH could readily acquire not only from the living environments but also from the host plant during feeding, we speculate that the bacterial community composition, vis-à-vis the relative abundance and diversity of *Pseudomonas* species, would vary across BPH populations collected from different geographical locations. Moreover, *Pseudomonas* represents one of the predominantly occurring phylum (i.e., the Gammaproteobacteria) in insects. Currently, more than 140 species of *Pseudomonas*, possessing remarkably different biology, are known to exist (Gomila et al., 2015). While some *Pseudomonas* species are capable of synthesizing essential amino acids and metabolites, others possess the capacity to detoxify xenobiotics (Silby et al., 2011). Therefore, considering the diverse traits of different *Pseudomonas* species, we focused on studying their dynamics in BPH. However, it is pertinent to first identify different species of *Pseudomonas* present in BPH and then, based on their identity, assign their likely role in BPH survival.

With this view, the current study was undertaken to identify changes in the BPH microbiome, with regard to *Pseudomonas*, in response to changing climates and geographical locations. Here, *Pseudomonas*-specific *16S rRNA* gut-microbiome profiling was carried out for investigating the diversity and abundance of *Pseudomonas* species present in the BPH populations collected over different seasons and from the various rice-growing regions in India. The insights obtained from this study provided a new perspective in understanding the role of certain microbes in the life history of BPH. Besides, being a primary symbiont, *Pseudomonas* can be engineered to effectively manage this pest, thereby offering alternate strategy(ies) for combating BPH outbreaks. Moreover, the information obtained can form the basis for monitoring different populations of BPH, thus serving as a promising approach for managing BPH populations as part of an effective strategy for integrated pest management (IPM) of this destructive pest of rice.

## Results

### Screening BPH populations for the presence of *Pseudomonas*

**Figure 1:**
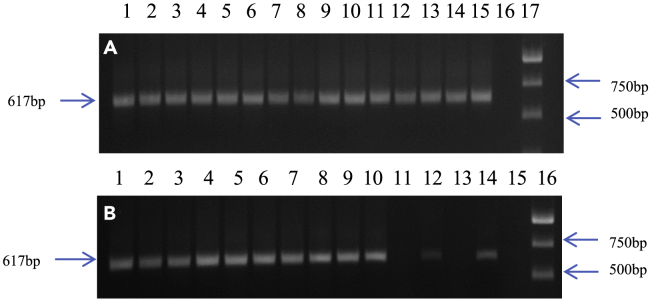
Screening BPH populations, collected from different rice-growing regions in India, for the presence of *Pseudomonas* (See ‘STAR Methods’ for details). A 0.8% agarose gel showing the diagnostic 618 bp PCR-amplified *Pseudomonas*-specific *16S rRNA* fragment (arrows on the left). (A) Lanes 1–5: Delhi; 6–10: Nalgonda1; 11–15: Nalgonda2; 16: Blank; 17: 1Kb DNA Ladder. (B) Lanes 1–5: Tripura; 6–10: Manipur; 11, 13 & 15: Blank; 12 & 14: Positive Control; 16: 1Kb DNA Ladder. Arrows on the right indicate the 500 bp and 750 bp fragments of the 1Kb ladder.

**Figure 2:**
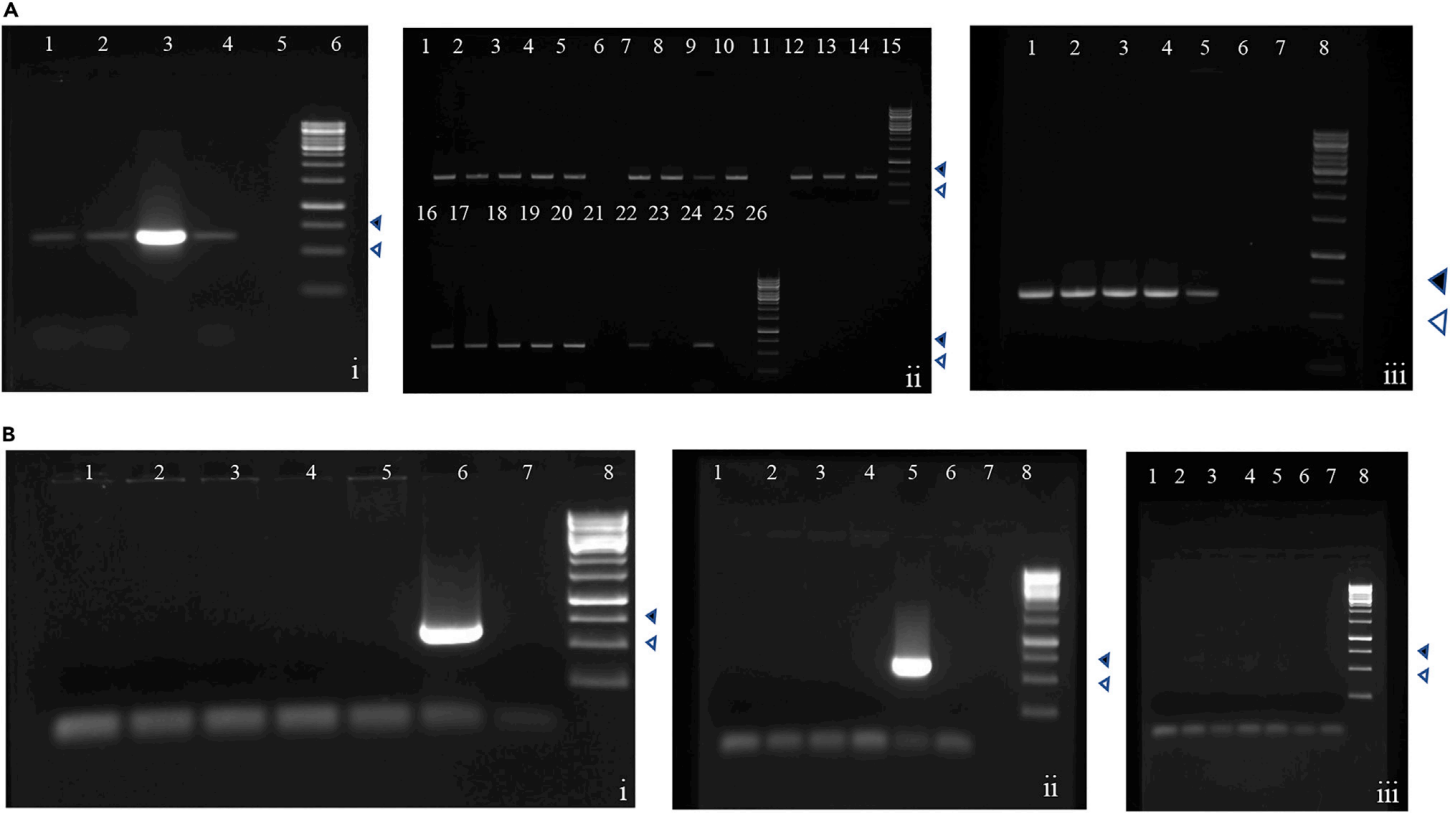
Seasonal fluctuations of *Pseudomonas* in the BPH populations collected in Delhi over a period of seven months (May–Nov) (See STAR Methods section for details). (A) 0.8% agarose gel showing the 618 bp PCR-amplified *Pseudomonas*-specific *16S rRNA* fragment. (i) Lanes 1–4: BPH individuals collected in May; Lane 5: Blank; Lane 6: 1Kb DNA Ladder; (ii) Lanes 1–5: DBOD individuals; Lanes 7–10 & 12–14: BPH individuals collected in June; Lanes 6 & 11: Blank; Lane 15: 1Kb DNA Ladder; Lanes 16–20: BPH individuals collected in November; Lanes 21, 23 & 25: Blank; Lanes 22 & 24: Positive control; Lane 26: 1Kb DNA Ladder; (iii) Lanes 1–5: BPH individuals collected in August; Lanes 6–7: Blank; Lane 8: 1Kb DNA Ladder. (B) 0.8% agarose gel showing the absence of the 618 bp PCR-amplified *Pseudomonas*-specific *16S rRNA* fragment in BPH individuals collected during the months of July, October, and September. (i) Lanes 1–5: BPH individuals collected in July; Lane 6: Positive control; Lane 7: No template control; Lane 8: 1Kb DNA Ladder; (ii) Lanes 1–4: BPH individuals collected in October; Lane 5: Positive control; Lane 6: No template control; Lane 7: Blank; Lane 8: 1Kb DNA Ladder; (iii) Lanes 1–7: BPH individuals collected in September; Lane 8: 1Kb DNA Ladder. The 500 bp () and 750 bp () fragments of the ladder are indicated.

PCR amplification of BPH genomic DNAs using primers specific for the hypervariable V3-V4 region of *Pseudomonas 16S rRNA* (see STAR Methods section) yielded a 618 bp fragment from BPH insects collected from the Southern (Nalgonda1 and Nalgonda2), Northern (Delhi; June, August, and November collections), and North-Eastern (Manipur and Tripura) regions (Figures 1 and 2A). However, this fragment failed to amplify in BPH insects collected in Delhi (the Northern region) during July, September, and October (Figure 2B)*.*

### Analysis of nanopore sequencing reads

Nanopore sequencing libraries constructed from PCR-amplified *Pseudomonas*-specific V3-V4 region of the *16S rRNA* fragment (618 bp) from BPH populations generated a total of 2,441,632 reads on the GridION (ONT) platform, of which 2,412,690 high-quality reads were retained after base calling, quality filtering, and adapter trimming for downstream analyses. These high-quality reads accounted for >96% of the total reads obtained for each sample (Table S1).

### Identification and classification of *Pseudomonas* species in BPH populations

**Figure 3:**
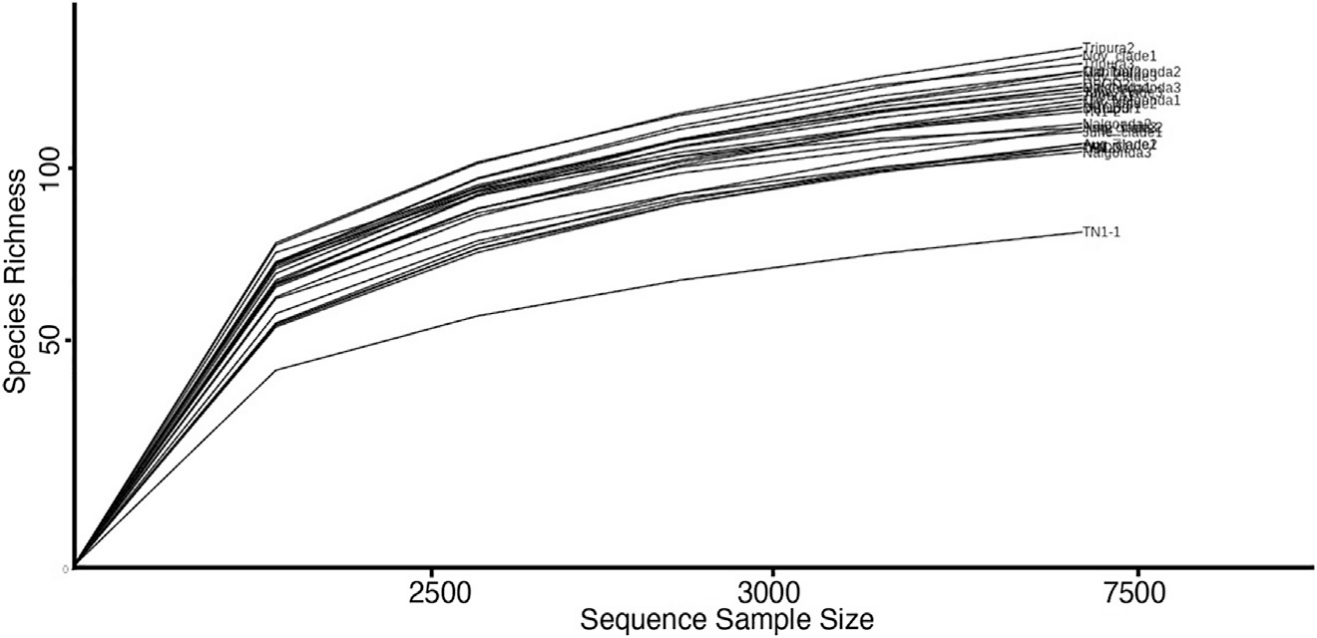
Comparative rarefaction curves indicating species richness of *Pseudomonas* in BPH populations and TN1 rice plants Owing to most samples showing similar rarefaction curves, individual sample labels are not discernible.

Of all the reads obtained, a total of 2,344,158 reads (97%) represented the phylum Proteobacteria. Of these, 2,235,316 reads belonged to Gammaproteobacteria, 2,066,727 were assigned to Pseudomonadales, and 1,964,565 belonged to Pseudomonadaceae. Finally, 1,692,489 could be classified as *Pseudomonas* (Table S2) with high accuracy. Species-level identification was carried out on the EPI2ME platform, where >95% reads were classified up to the species level with >88% accuracy. The rarefaction curve analysis indicated that the sequencing depth and coverage were good (Figure 3).

### Data pre-processing and filtering

After taxonomic classification up to the species level, 861 taxa were identified across 27 samples, of which 182 OTUs with counts ≤2 were filtered out. 501 low-abundance taxa were removed based on prevalence, and 18 low-variance taxa were removed based on inter-quartile ranges. Finally, after the data filtering step, a total of 160 taxa (representing different species of *Pseudomonas* predominantly found in BPH) were retained for further analyses (Table S3).

### Diversity analyses

**Figure 4:**
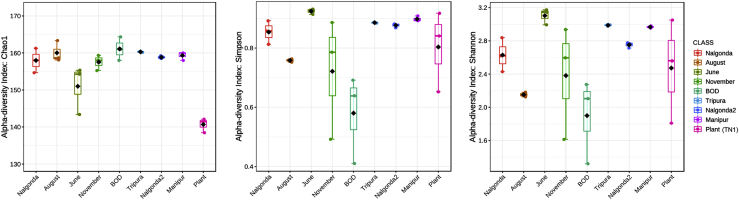
Boxplots representing the alpha diversity distribution of *Pseudomonas* sp. across BPH populations and TN1 rice plants *Pseudomonas* sp*.* richness and diversity were measured using Chao1, Simpson, and Shannon indices, respectively.

Post data normalization (see STAR Methods), the diversity of *Pseudomonas* species within and between samples was estimated by alpha- and beta-diversity analyses, respectively. The alpha diversity measured using species richness estimator (Chao1) and diversity indices (Shannon and Simpson) was plotted across 27 samples and reviewed as boxplots (with each boxplot representing the diversity distribution of a population; Figure 4). The species richness values ranged from 138 to 164 between samples, with TN1 (rice plants) showing the lowest value. However, minimal variation was observed across BPH populations, implying that the number of *Pseudomonas* species detected across the BPH populations assayed in this study was nearly equal. This was further supported by the rarefaction curve analysis, where the least number of OTUs were observed for TN1 plants. In contrast, the observed number of OTUs for BPH populations did not show much variation (Figure 3). However, while the species richness (number of OTUs) values (Chao1) showed a uniform distribution across samples, the Shannon and Simpson diversity index values differed significantly (Table S4). These values ranged from 1.32 to 3.17, and the values of the Simpson diversity index varied from 0.41 to 0.93 (Table S4), indicating differences between BPH populations with regard to the proportion of species abundance (evenness) within samples.

**Figure 5:**
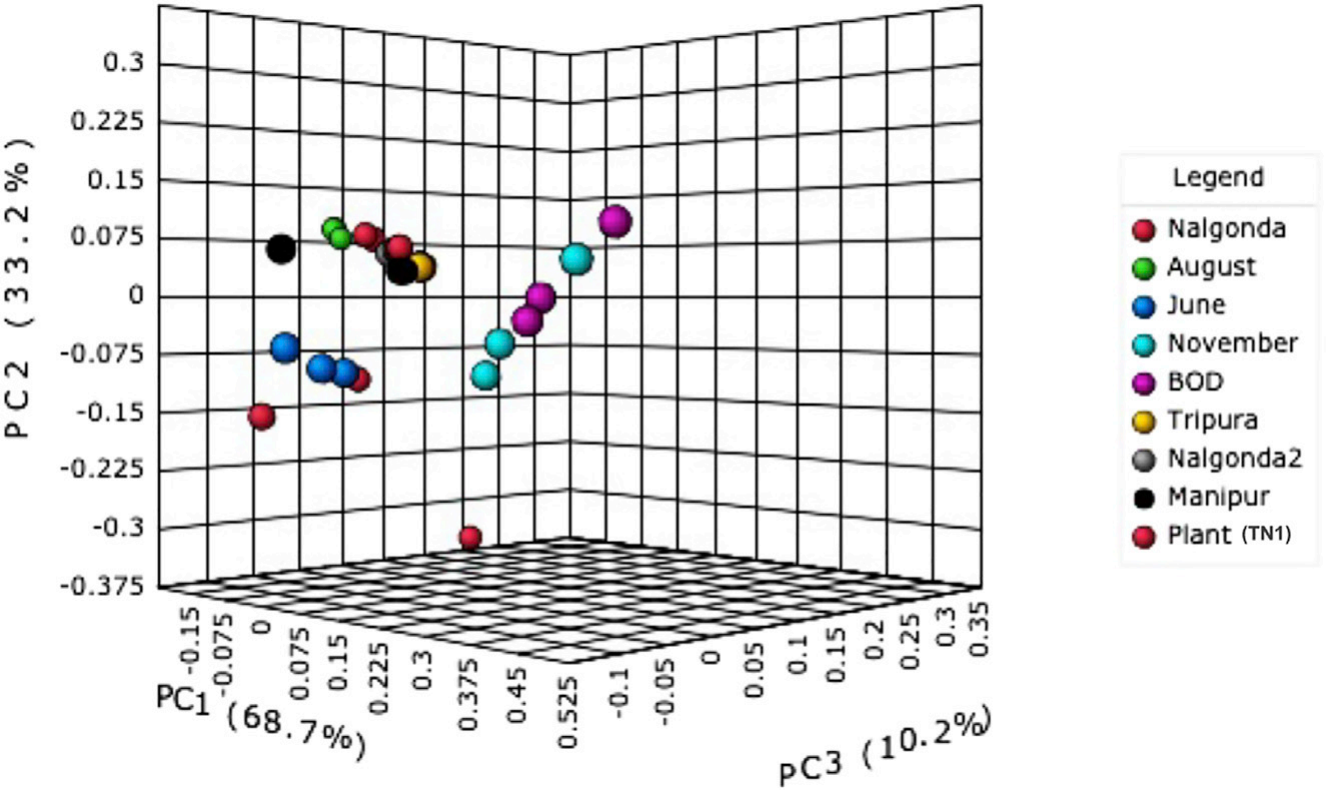
Beta diversity distribution of *Pseudomonas* sp. across BPH populations and TN1 rice plants using 3D principle coordinate analysis (PCoA) plots with each axis representing variation between samples (p-value cut-off ≤ 0.001).

Furthermore, the beta-diversity analysis revealed variation in species diversity and composition between samples when viewed using 3D principal coordinate analysis (PCoA) plots with each axis representing the variation between samples. The X axis (PC1) represented the highest dimension of variation (68.4%), followed by the Y axis (PC2), representing the second-highest dimension of variation (33.1%) and Z axis (PC3), indicating the third-highest dimension of variation (10.1%) (Figure 5). Additionally, PERMANOVA analysis [R-squared 0.8725, p value < 0.001] carried out to assess the statistical significance of the clustering pattern in the ordination plot (Figure 5) revealed that the estimated distance values, calculated based on the Jenson-Shannon divergence index were significant. Taken together, it was observed that the BPH collected from different rice-growing regions of India had varied microbiomes with regard to the relative abundance and prevalence of *Pseudomonas* species.

### Identification of differentially abundant species

**Figure 6:**
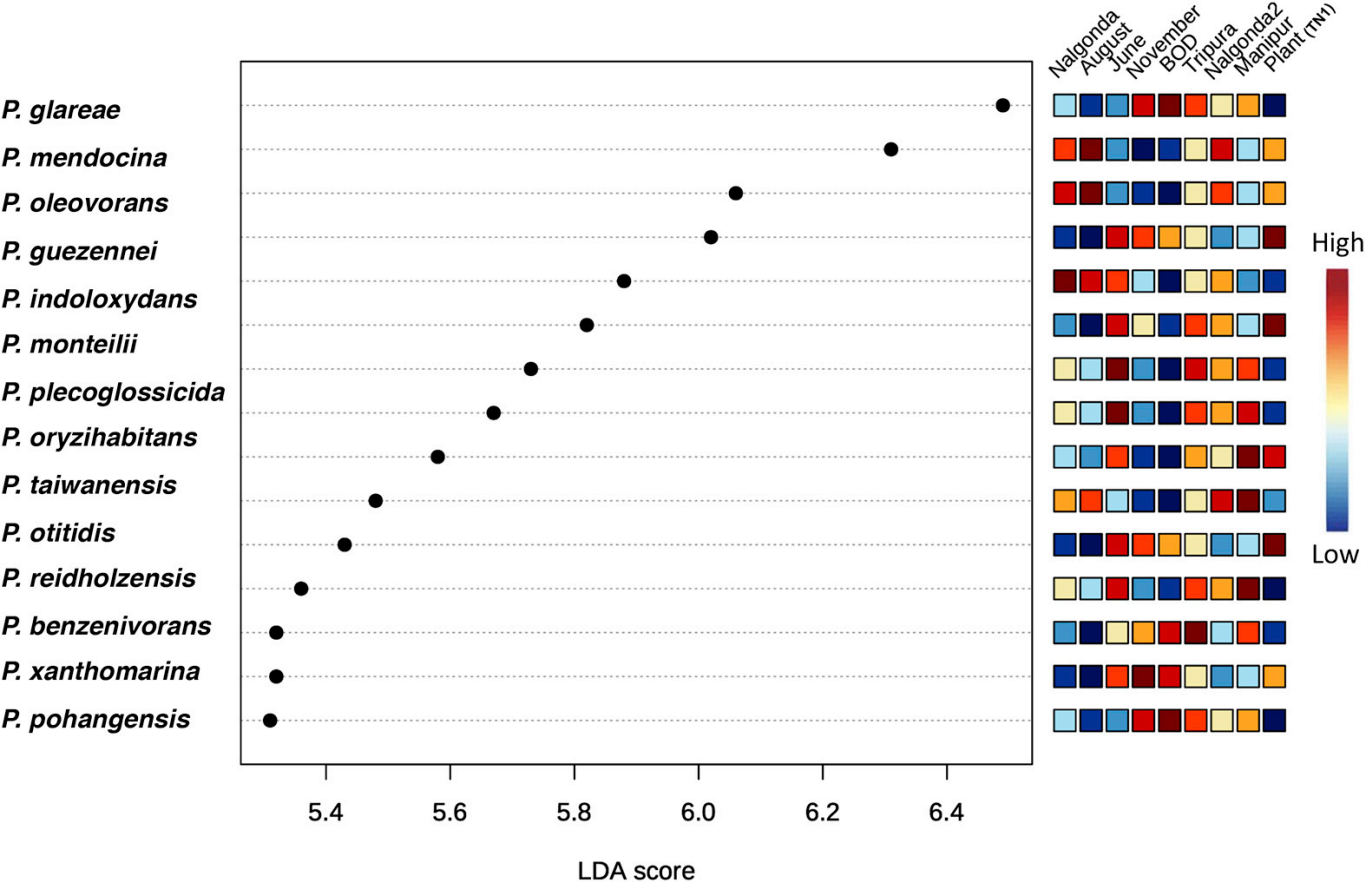
Identification of differentially abundant *Pseudomonas* species across BPH populations based on linear discriminant analysis effect size (LEfSe) along with their linear discriminant analysis (LDA) score (p-value cut-off ≤ 0.05).

DESeq2 variance estimation identified the top 50 species of *Pseudomonas* that showed differential abundance across BPH populations (Table S5), and some of those identified species being *P.* *indoloxydans, P.* *plecoglossicida, P.* *glareae, P. fluvialis, P.* *monteilii, P.* *otitidis, P.* *stutzeri,* and *P.* *guezennei.* Most of the species identified by DESeq2 analysis concurred with those identified by the univariate analysis (Table S6), thereby confirming the relevance and reliability of the data. Furthermore, the LDA (Linear Discriminant Analysis) effect size (LEfSe) analysis identified >15 species of *Pseudomonas* with a significant effect size (Figure 6). *Pseudomonas* species that showed maximum variation between BPH populations (with LDA score >5.8) were *P. glareae, P.* *mendocina, P.* *oleovorans, P. guezennei, P. indoloxydans,* and *P. monteilii.*

### Identification of abundant and core *Pseudomonas* species

**Figure 7:**
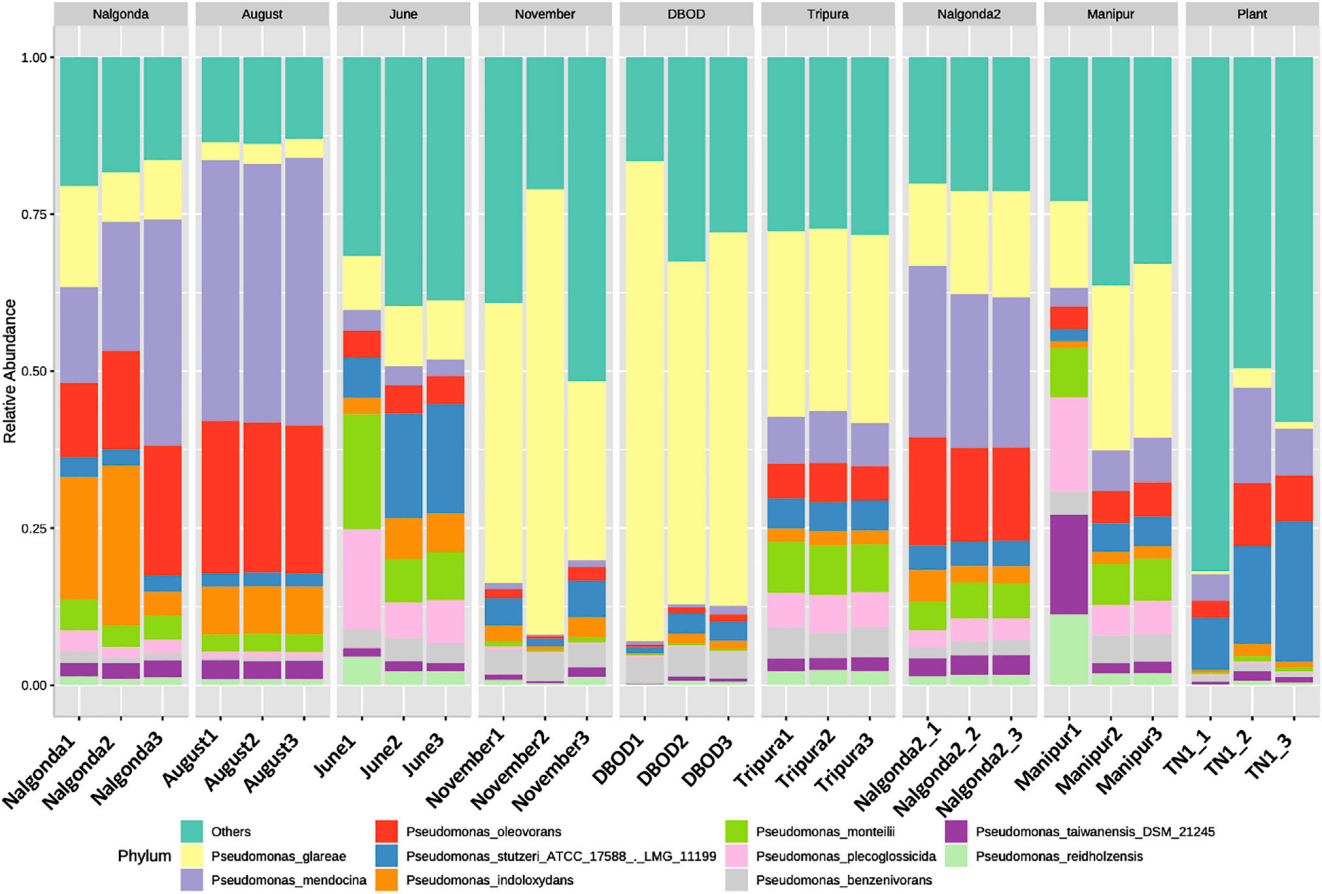
Stacked bar plots depicting the variation in *Pseudomonas* species composition, their relative abundance and diversity between BPH populations and TN1 rice plants

**Figure 8:**
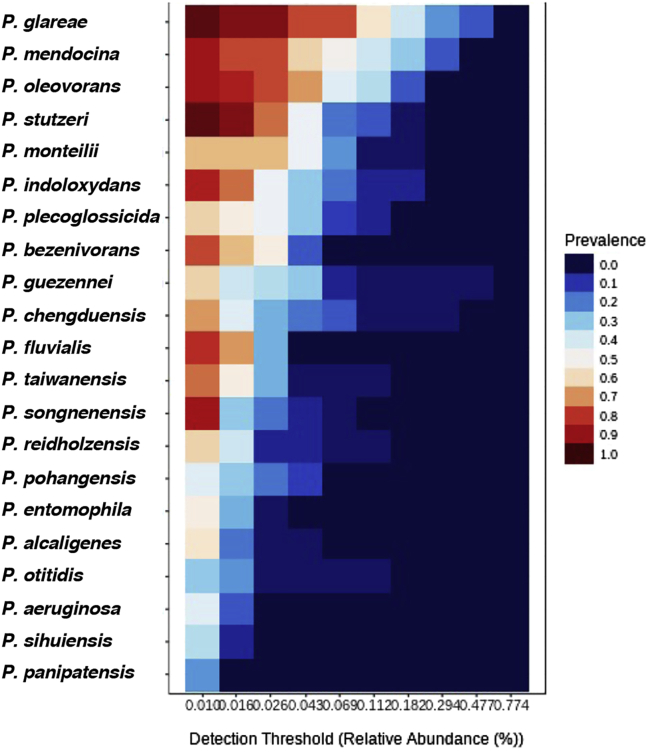
Core microbiome analysis with reference to *Pseudomonas* sp. across BPH populations Heatmap representing their relative prevalence across BPH populations is shown with blue and red signifying the lowest and the highest values, respectively. The *x* axis represents the detection thresholds (indicated as relative abundance) from lower (left) to higher (right) abundance values. Color shading indicates the prevalence of each *Pseudomonas* species among samples for each abundance threshold. As we increase the detection threshold, the prevalence decreases.

Variation in species composition, their relative abundance, and diversity between BPH populations was evident from the stacked bar plots (Figure 7). While *P. glareae, P. plecoglossicida,* and *P. monteilii* were predominantly present in the North-Eastern BPH populations, *P. mendocina, P. oleovorans, P. indoloxydans,* and *P. stutzeri* were found in abundance in the Southern populations. BPH individuals collected in Delhi during August (over a period of two years) showed high resemblance to the Southern population, especially with regard to the titers of *P. mendocina* and *P. oleovorans*, while the Delhi population was represented by insects collected during June (for two years) shared similarities with the North-Eastern population, particularly in terms of titers of *P. plecoglossicida* and *P. monteilii*. The November-collected insects and the BOD-cultured BPH had extremely high titers of *P. glareae* followed by *P. guezennei* while the other species were present in insignificant amounts. *Pseudomonas* present in TN1 plants was primarily represented by *P.* *chengduensis, P. guezennei,* and *P. stutzeri,* along with various other species of *Pseudomonas* in very low titers. Furthermore, over 20 species of *Pseudomonas* were identified that were ubiquitously present (irrespective of their relative abundance) in all the BPH populations, as revealed by the results obtained from core microbiome analysis (Figure 8, Table S7).

### Hierarchical clustering analysis

**Figure 9:**
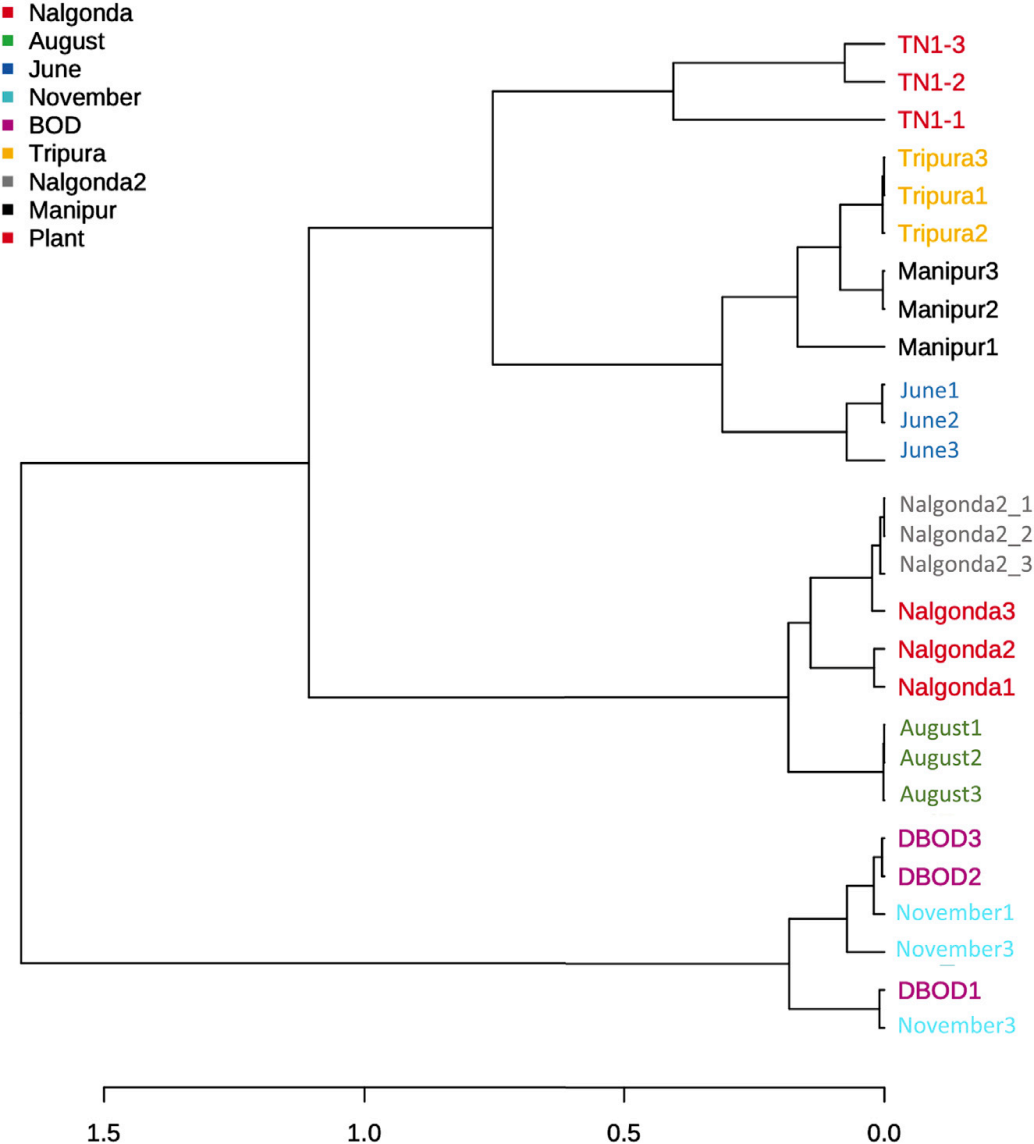
Hierarchical clustering analysis (based on the presence and abundance of different *Pseudomonas* sp.) performed for BPH populations and TNI rice plants For clustering, the distance estimations based on Jaccard index and Ward’s linkage algorithm was used.

Based on *Pseudomonas* species composition, diversity, and relative abundance across samples, hierarchical clustering analysis grouped BPH populations into four discrete clusters. BPH insects found in Delhi during June shared higher similarity with the North-Eastern populations (Manipur and Tripura) and were clustered together, whereas the insects collected during August were more closely related to the Southern populations (Nalgonda1 and Nalgonda2) and thus formed a separate clade (Figure 9). Interestingly, the BOD incubator-collected BPH insects (DBOD) were grouped with the November-collected insects, while TN1 plants formed a separate cluster (Figure 9).

### Estimation of *Pseudomonas* titers in pesticide-exposed insects

**Figure 10:**
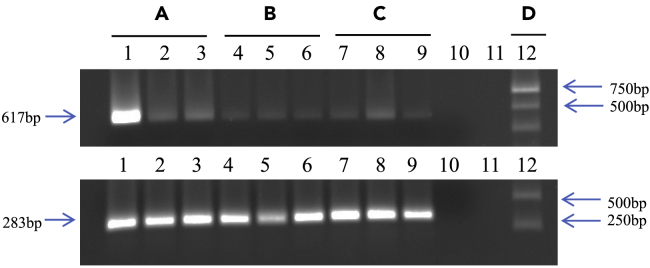
Effect of pesticide (imidacloprid) on *Pseudomonas* titers in BPH as revealed by semi-quantitative PCR Individuals from pesticide-exposed and un-exposed (control) BPH populations were assayed using semi-quantitative PCR with *Pseudomonas*-specific *16S rRNA* primers to amplify a 618 bp fragment (for details see ‘STAR Methods’). Samples assayed were BPH individuals from a population (A) exposed to pesticide (1^st^ generation; lanes 1–3) (B) exposed to pesticide (4^th^ generation; lanes 4–6) (C) unexposed to pesticide (control; lanes 7–9) for over 23 generations (D) 1Kb DNA Ladder. The semi-quantitative PCR products were separated on a 1.0% agarose gel. Lanes 1–9: amplification of the *Pseudomonas*-specific *16S rRNA* fragment from BPH individuals as indicated above the lanes; Lanes 10–11: Blank; Lane 12: 1Kb ladder. Figures and arrows on the left indicate the PCR amplified fragments and their respective sizes (618 bp *Pseudomonas*-specific *16S rRNA* fragment and the 283 bp *Actin* fragment). The lower panel represents PCR amplification controls (*Actin*; see ‘STAR Methods’ for details) for the respective lanes on the upper panel. Arrows and figures on the right indicate the 750, 500, and 250 bp fragments of the 1Kb ladder.

Approximately, a 2-fold increase in *Pseudomonas* titers was observed in BPH after exposure to pesticide (first generation under pesticide stress). However, the numbers were restored to pre-exposure levels by the 4^th^ generation (Figure 10), i.e., 4^th^ generation pesticide-exposed BPH and BOD-cultured insects had similar titers of *Pseudomonas*.

## Discussion

Seasonal long-distance movements of the BPH have been well-documented through meteorological assays and radar observations (Pender, 1994; Qi et al., 2014; Hereward et al., 2020). While it is well established that BPH traverses thousands of kilometers, encountering and surviving diverse environmental and nutritional stresses during its migration, the mechanisms that endow BPH with the capacity to quickly adapt and survive harsh environments remain largely unknown. Owing to the diverse roles and contributions of gut microbes toward insect fitness and longevity, the gut microbiome could act as a major key player in mediating BPH’s rapid adaptive responses to stress. Bacterial community structure and diversity, especially for migratory insects, are largely influenced by the host plant and feeding/collection site, as bacteria constituting the microbiome are continuously being taken up by insects as they interact with their environment (Jones et al., 2019). Previous studies have indicated the dynamic nature of the BPH microbiome, where it has been shown that not only does the composition of bacterial species vary between BPH populations feeding on different rice varieties but also between biotypes (Tang et al., 2010). However, there is very little information with regard to the alterations in the relative abundance or proportion of different bacterial species within BPH, in response to the changing environmental conditions, that likely account for the observed variation in virulence and differential response of insect populations to biotic and abiotic stresses. Hence, the present study was initiated to assess the rapid shifts in the BPH microbiome structure and composition in response to the external environment.

Taking into account the diverse traits and roles undertaken by *Pseudomonas* sp. as a component of the insect microbiome, we focused on assessing the diversity and abundance of *Pseudomonas* sp*.* across BPH populations collected from different rice-growing regions of India. While *Pseudomonas* was ubiquitous in the BPH populations collected from the North-Eastern and the Southern parts of India, insects collected from the Northern region (Delhi) showed differential presence across seasons. The Delhi individuals collected during June, August, and November were positive for *Pseudomonas*, whereas BPH found during May, July, September, and October were negative for *Pseudomonas* (Figure 2B). This suggests that (i) different BPH populations with regard to their microbiome composition are present in Delhi, or (ii) BPH keeps rapidly altering its gut microflora in response to the external environmental conditions.

In support of our first explanation, it is worth noting that the Northern regions of India experience extremely harsh winters with temperatures dipping to less than 5°C, which is apparently lethal for BPH (Srinivasa et al., 2020). Moreover, in the Northern region, rice is primarily sown during May–June and harvested during October–November, and as a result, rice host is not available in the North for the monophagous BPH to feed on throughout the year. However, in the Southern and the North-Eastern parts of India, rice is extensively cultivated throughout the year, and therefore, a high likelihood that BPH overwinters in these regions. Moreover, in the Southern region, the temperatures are much more favorable for BPH survival, especially during the winter months. Interestingly, when taken together, these observations imply that BPH populations found in Delhi during May–June likely originated from BPH populations that have migrated from distant regions rather than from populations present locally, if any. Moreover, due to its small bodyweight, BPH can easily ride the prevailing wind currents, and therefore, the BPH populations in Delhi are likely to be influenced by the direction of prevailing wind currents. Collectively, this explains the probability of finding different BPH populations in Delhi as a consequence of the continuous inflow of BPH insects across seasons.

However, the second statement is reinforced by the possibility that BPH migrates to Delhi during May–June and reproduces for two-three generations before the rice crops reach maturity by early- to mid-August. And being an “r-strategy” organism (Hu et al., 2014), BPH population size increases exponentially, which consequently leads to an outbreak. Therefore, the fluctuations observed in microbiome composition of BPH individuals sampled in Delhi during the remaining months (after May–June) of the year are probably not due to sampling of individuals of different migratory BPH populations but are rather due to a strategy deployed by the dominant BPH population (likely an offshoot of the population(s) that has/have already migrated to Delhi during May–June) to successfully colonize and adapt to the external environmental conditions and different rice varieties.

Indications of rapid shifts in the microbiome structure of BPH come from studying the *Pseudomonas* diversity across BPH populations collected from different rice-growing regions of India. While the number of OTUs (corresponding to different *Pseudomonas* species) identified from BPH populations (analyzed in the current study) was nearly the same (Figure 4), these populations differed in the relative abundance (prevalence) of each *Pseudomonas* species (Table S4; Figure 5). The implication of this observation being that though the “core” microbiome undergoes minimal changes with regard to its composition (presence/absence of any bacterial species) in response to the environment, the relative amounts of these bacterial species change considerably and thereby hinting at the involvement of microbiome in facilitating rapid adaptive responses in BPH.

At this juncture, we suppose that the numbers of a particular *Pseudomonas* species in BPH are primarily determined by its role and functional capabilities (Table S8). And this is supported by the data obtained from identifying differentially abundant species of *Pseudomonas* across BPH populations (Figure 7). Here, we observed that most of the *Pseudomonas* species identified (by DESeq2 variance estimation and univariate analysis) in BPH are either capable of xenobiotic detoxification or possess sugar assimilation and nitrate reduction capacity (Table S5). For instance, *P. glareae, P. plecoglossicida,* and *P. monteilii*, which are present in high abundance in the North-Eastern BPH populations, are largely capable of heavy metal detoxification, nitrate reduction, and protein assimilation (Romanenko et al., 2015; Nishimori et al., 2000; Elomari et al., 1997); whereas, *P. mendocina, P. oleovorans, P. indoloxydans,* and *P. stutzeri,* which are predominantly found in the Southern populations, mainly possess xenobiotic detoxification capacity (Manickam et al., 2008; Mir et al., 2017; Itoh et al., 2018). The extensive usage of pesticides in paddy cultivation to augment the agricultural output and manage BPH outbreaks leaves the insect with no choice but to evolve mechanisms to resist xenobiotics. And as reported by Devi et al. (2017), the majority of areas in the Southern region apply higher levels of pesticides as compared to the North-Eastern region; therefore, it is plausible that insects collected from the South (Nalgonda1 and Nalgonda2) carry higher titers of these bacterial species which can potentially assist in pesticide degradation.

Further, while recruiting certain species of *Pseudomonas* can favor BPH survival under pesticide stress, adopting others could substantially enhance its fitness. It has been observed by Rashid et al. (2017) that nitrogenous fertilizers add to the fitness of BPH by increasing its chances of survival and developmental rate. Therefore, it seems practical for BPH to maintain high titers of bacterial species capable of nitrate reduction as observed for the North-Eastern population (Figure 7), where the addition of nitrogenous fertilizers is common practice (Sivagnanam and Murugan, 2019). Collectively, these observations substantiate our hypothesis that BPH adjusts the quality and quantity of bacterial symbionts within its gut, depending on the requirement.

This is further supported by the data on species diversity and abundance pattern of *Pseudomonas* in BPH populations collected from Delhi across different seasons. Considering the fact that in Delhi, the rice crop is primarily sown around May–June and the application of nitrogenous fertilizers in paddy fields during the early stages of rice cultivation is comparatively higher, therefore, as predicted, higher titers of nitrate-reducing bacteria, i.e., *P. plecoglossicida* and *P. monteilii,* were observed in BPH populations collected in June*.* Similarly, the microbiome of the BPH found in Delhi in August resembles that found in the Southern BPH population, with regard to the titers of pesticide-degrading bacteria, particularly *P. mendocina* and *P. oleovorans.* It is worth noting that during the time frame in which the rice crops attain maturity, the external climatic conditions (such as temperature, rainfall, and humidity) becomes optimum for BPH proliferation as well, leading to a massive upsurge in the BPH populations and a consequent increase in the usage of pesticides for controlling BPH outbreaks in the paddy fields. Hence, the observed higher titers of detoxifying *Pseudomonas* species in BPH populations, collected in August, are probably a strategy adopted to help them tolerate/resist these pesticides.

Furthermore, the proportion of *Pseudomonas* species found in the November-collected insects resembled DBOD insects (lab-grown BPH population). One likely reason for this observation could be the existence of a physiological trade-off or fitness cost associated with the ability of insect hosts to modulate their gut microbial community dynamics in response to the environment. Generally, early- to mid-November not only marks the onset of the end of the rice-growing season in the Northern parts of India but is also the time when the BPH population in Delhi starts to decline. Food scarcity coupled with the dipping ambient temperature compels BPH to enter its migratory phase. Thus, BPH would need to judiciously reallocate its energy resources toward migration rather than feeding and reproduction. And as a result, to reduce fitness costs, the titers of different *Pseudomonas* species in the November-collected insects were restored to the levels observed for BOD-cultured insects. Additionally, the observed seasonal fluctuations in the microbial titers could also be an outcome of changing external temperatures. In fact, a recent study (Zhang et al., 2021) has demonstrated the effect of temperature on BPH-bacterial symbiosis and its consequent impact on host susceptibility to insecticide (imidacloprid).

Although the process that drives microbial shifts in BPH in response to the environment remains largely unknown, a trade-off with the immune response is one possibility. Perhaps, the alterations in the immune status in resistant/tolerant insects inhibit the growth of some symbionts while allowing others to proliferate as observed in *Drosophila*, *Aedes*, and *Anopheles* (Douglas, 2014; Pietri and Liang, 2018; Gupta and Nair, 2020). Moreover, as the *Pseudomonas* represents a “transient” component of the BPH microbiome that could be readily acquired during the feeding process, its composition would also depend on the rice plant variety it is feeding upon. Several species of *Pseudomonas,* such as *P. chengduensis, P. guezennei,* and *P. stutzeri,* detected in BPH were also found to be present within the un-infested TN1 rice plants. Acquisition of these bacterial species by BPH during the feeding process could directly influence its microbiome. Besides, the host plants can also regulate the titers of bacterial symbionts in insects by modulating the insect metabolism and thereby determining the nutrient availability for these commensal microbes. However, further studies corroborating this hypothesis are required.

The data obtained from the semi-quantitative PCR analyses (Figure 10) indicated that selection using the pesticide (imidacloprid) induced significant changes in the relative abundance of *Pseudomonas* in BPH. We observed more than 2-fold increase in the *Pseudomonas* titers immediately after exposure to the pesticide, which was restored to pre-exposure levels by the fourth generation. This further suggests that the ability of insects to alter their gut microflora likely carries a fitness cost. Moreover, it also indicates that modulation of bacterial symbionts is predominantly a quick response of insects to combat/alleviate immediate stress rather than a long-term resistance strategy. As the titer of individual symbiont species and the overall composition of the microbiome can evolve rapidly in the BPH population in response to the environment, it could potentially contribute to BPH’s rapid adaptive response to stress. And although the relative titer of bacterial species eventually returns to its pre-pesticide exposure levels, it could provide BPH with enough time to initiate other stress-related pathways that enable it to resist/tolerate the stress. However, additional experiments are required to validate this hypothesis. Besides, it is worth mentioning that the insect-microbiome interaction is a very complex process; where, in addition to the effects of individual members of the microbiome on hosts, microbial symbionts may themselves form communities and influence each other (Ferrari and Vavre, 2011), thereby implying that *Pseudomonas* species alone may not aid BPH gain adaptive traits. Therefore, future studies aimed at deciphering such interactions and mechanisms underlying pesticide degradation and understanding the contributions of bacterial symbionts toward these processes are warranted.

Taken together, the data from the present study indicate that the changes in the microbial community within BPH are correlated with the environmental fluctuations and, therefore, add to our existing knowledge of symbiont-mediated insect adaptation. And understanding the implication of these changes in *Pseudomonas* species composition across BPH populations could eventually help us restrict the BPH population size, as *Pseudomonas*, being a primary symbiont, can be engineered as a part of an effective strategy for managing this destructive pest of rice. In addition, the present study can also form the basis for utilizing symbiont-based approaches to monitor different populations of BPH and provide farmers with an early warning as to the choice of pesticides during the different periods of rice cultivation.

### Limitations of the study

The current study highlights the microbial community dynamics (determined by monitoring shifts in *Pseudomonas* species composition) within BPH and suggests the likely involvement of gut microbes in facilitating its survival under rapidly changing environmental conditions. Variation in the *Pseudomonas* species composition and abundance across BPH populations is indicative of a remarkably complex interplay between insect physiology and the microbial community, which likely endow BPH with a capacity to respond rapidly to resist/tolerate stress. However, a more detailed exploration is necessary to fully understand and determine the extent to which microbial community dynamics regulate BPH’s responses to stress. Besides, the fact that bacterial symbionts act in synergy to influence ecologically important traits of their insect host cannot be overlooked. It is often observed that several bacterial strains or species co-occur within the host and the presence of multiple species is required for the insect survival. Hence, it is possible that several bacterial symbionts, and not just *Pseudomonas*, are contributing toward pesticide resistance and helping BPH overcome environmental stresses. Therefore, a complete understanding of multi-species symbiont communities within insects and their possible contributions toward host survival needs to be investigated. Further, it is also crucial to decipher the molecular processes that drive microbial shifts in BPH in response to environmental fluctuations. Moreover, while we suggest that *Pseudomonas* species composition and abundance is dependent on their role and function within BPH, a deeper investigation into understanding the implication of such changes is required to ascertain its involvement in mediating rapid adaptive responses of BPH to changing climates and geographical locations.

## STAR★Methods

### Key resources table

**Table:** 

REAGENT or RESOURCE	SOURCE	IDENTIFIER
**Biological samples**		

Brown planthopper (BOD population)	New Delhi, India	DBOD
Brown planthopper (June population)	New Delhi, India	June
Brown planthopper (August population)	New Delhi, India	August
Brown planthopper (November population)	New Delhi, India	November
Brown planthopper (Manipur population)	Manipur, India	Manipur
Brown planthopper (Tripura population)	Tripura, India	Tripura
Brown planthopper (Nalgonda population; 2015)	Nalgonda, India	Nalgonda1
Brown planthopper (Nalgonda population)	Nalgonda, India	Nalgonda2
Rice, *Oryza sativa*	TN1 variety	TN1

**Chemicals, peptides, and recombinant proteins**		

Deoxynucleotide (dNTPs) Solution Mix	New England BioLabs, USA	Cat#N0446S
Taq DNA polymerase	BangaloreGeNei, India	Cat#0601600051730
Imidacloprid (Confidor 17.80% SL)	Bayer AG, Germany	N/A
Gene ruler 1Kb DNA ladder	Thermo Scientific, USA	Cat#SM0311
Vermiculite	AM Biotech, India	N/A

**Critical commercial assays**		

GF-1 Tissue DNA Extraction kit	Vivantis, Malaysia	Cat#GF-TD-050
GeneJET Plant Genomic DNA Purification kit	Thermo Scientific, USA	Cat#K0791
Qubit dsDNA BR Assay kit	Invitrogen, USA	Cat#Q32853
GF-1 AmbiClean kit (Gel & PCR)	Vivantis, Malaysia	Cat#GF-GC-100
NEBNext Ultra II DNA Library Prep Kit for Illumina	New England BioLabs, USA	Cat#E7645L
PCR Barcoding Expansion 1–96 kit	Oxford Nanopore Technology, UK	Cat#EXP-PBC096
Ligation Sequencing Kit	Oxford Nanopore Technology, UK	Cat#SQK-LSK109

**Deposited data**		

Sequence Read Archive (SRA) files	NCBI, https://www.ncbi.nlm.nih.gov/	GenBank: PRJNA733325

**Oligonucleotides**		

Pseudo-S2-F	5′-GACGGGTGAGTAATGCCTA-3′	Spilker et al. (2004)
Pseudo-S2-F	5′-CACTGGTGTTCCTTCCTATA-3′	Spilker et al. (2004)
ACT-mod F	5′-TGCGTGACATCAAGGAGAAGCTG-3′	This study
ACT-mod R	5′-GTACCACCGGACAGGACAGT-3′	This study

**Software and algorithms**		

MinKNOW 2.1 v18.05.5	Oxford Nanopore Technology, UK	N/A
Albacore v2.3.1.	Oxford Nanopore Technology, UK	N/A
EPI2ME Agent Software	Oxford Nanopore Technology, UK	N/A
ranacapa R package	Kandlikar et al. (2018)	N/A
MicrobiomeAnalyst Software	https://www.microbiomeanalyst.ca/	N/A
Phyloseq R package	McMurdie and Holmes (2013)	N/A
R package microbiome	http://microbiome.github.io	N/A
Image Lab software v6.0.1	Bio-Rad Laboratories, USA	N/A

**Other**		

Qubit 4.0 fluorometer	Invitrogen, USA	Cat#Q33226
Gel Documentation System - Alpha Imager HP	Protein Simple, USA	Cat#921382400
NanoVue Plus Spectrophotometer	GE Healthcare, UK	Cat#28-9232-15
GridION X5	Oxford Nanopore Technology, UK	Cat#GRD-X5B003
SpotON flow cell R9.4	Oxford Nanopore Technology, UK	Cat#FLO-MIN106D
BOD Incubator	Hicon Eminence, India	N/A
Veriti 96 well thermal cycler	Applied Biosystems, USA	Cat#4375786

### Resource availability

#### Lead contact

Further information and requests for resources, reagents and strains should be directed to and will be fulfilled by the lead contact, Suresh Nair (suresh@icgeb.res.in).

#### Materials availability

This study did not generate new unique reagents. Insect and plant samples used in this study are available from the lead contact with a completed Materials Transfer Agreement.

### Experimental model and subject details

#### BPH populations

A total of 12 BPH populations were screened for the presence of *Pseudomonas*. Of these, two samples represented BPH collected from rice fields (during a major BPH outbreak in 2017) in the North-Eastern regions of India [one from Manipur (24.63° N, 93.75° E) and the other from Tripura (24.25° N, 92.15° E)]; two samples were collected during a BPH outbreak in the Southern region [(Nalgonda district, Hyderabad; 17.05° N, 79.26° E) designated as Nalgonda1 (collected in 2015) and Nalgonda2 (collected in 2017)] and seven samples represented BPH populations found in Delhi (28.52° N, 77.16° E), collected over a period of two consecutive years (2016–2017) in the months of May-November. One BPH population (referred to as DBOD) was reared on TN1 (Taichung Native 1; a susceptible rice variety) in a BOD incubator (maintained at 28°C with 16 h light and 8 h dark photoperiod at ICGEB, New Delhi) for 15 generations and was used as control. All collected insects were preserved in absolute ethanol (99.9%) and stored at −20 °C till further use.

#### Plant samples

Regions near the base of the rice stem (which represented the active feeding site of BPH) were dissected from un-infested 15-day-old TN1 rice seedlings, which were grown in sterile vermiculite. The tissue was preserved in absolute ethanol and stored at −20°C till further processing.

### Method details

#### Genomic DNA extraction from rice and BPH

Total DNA was extracted from individual insects of each population using the GF-1 tissue DNA extraction kit (Vivantis, Malaysia) and from TN1 rice seedlings using the GeneJET Plant Genomic DNA Purification mini kit (Thermo Scientific, USA). The extracted genomic DNA was quantified on the Qubit 4.0 fluorometer (Invitrogen, USA). Further, the DNA quality was accessed by gel electrophoresis (using 0.8% TBE agarose gel; Maniatis et al., 1982).

#### Screening BPH populations for the presence of *Pseudomonas*

The *Pseudomonas-*specific V3-V4 hypervariable region of *16S rRNA* (for species-level identification) was PCR amplified using BPH DNA as a template and primer pair Pseudo-S2-F 5′-GACGGGTGAGTAATGCCTA-3' and Pseudo-S2-R 5′-CACTGGTGTTCCTTCCTATA-3′ (Spilker et al., 2004). The PCR amplification profile was initial denaturation at 95°C for 5 min, followed by 30 cycles of 95°C for 30s, 58°C for 1 min, and 72°C for 45s, and a final extension of 72°C for 5 min. 20 μl of PCR reaction contained 200 μM dNTPs, 0.6 U Taq DNA polymerase (Bangalore Genei, (India) Pvt. Ltd.), 1X Taq buffer, and 13 μM of each primer. The PCR amplified products were separated on 0.8% agarose gel.

#### Construction of *Pseudomonas*-specific *16S rRNA* library and sequencing

The PCR-amplified fragments (618 bp) (obtained from the BPH populations which were positive for *Pseudomonas*) were gel purified using GF-1 AmbiClean (Gel & PCR) kit (Vivantis, Malaysia). The eluted products were quantified using NanoVue Plus Spectrophotometer (GE Healthcare, UK) and sequenced using the Oxford Nanopore Technology (ONT, UK) by M/s Genotypic Technology Pvt. Ltd. (Bengaluru, India).

For library preparation, approximately 300 ng of the eluted PCR product, representing each amplicon, was end-repaired using the NEBnext ultra II end repair kit (New England Biolabs, MA, USA), and the reaction was cleaned up with 1x AmPure beads (Beckmann Coulter, USA). Next, the barcoding adapter ligation (BCA) was carried out using the NEB blunt/TA ligase (New England Biolabs, MA, USA) and cleaned with 1x AmPure beads. The barcoding adapter-ligated DNA was quantified using a Qubit 4.0 fluorometer (Invitrogen, USA). Further, these quantified, adapter-ligated amplicons were barcoded through PCR using the corresponding barcode primers and LongAmp Taq polymerase (LongAmp Taq 2x New England Biolabs, MA, USA), the reaction mixture for each amplicon was cleaned up with 1.6x AmPure beads (Beckmann-Coulter, USA). Purified barcoded amplicons were pooled in equal proportions from all the barcoded samples. The pooled sample was further end-repaired using the NEBnext ultra II end repair kit and cleaned up with 1x AmPure beads. The end-repaired amplicons were ligated with 1D Adapter using NEB blunt/TA ligase (New England Biolabs, MA, USA) and cleaned up using 0.4x Ampure beads (Beckmann Coulter, USA). The library was eluted in 16 μl of elution buffer and was used for sequencing. The barcoding and library preparation was performed using EXP-PBC096 and SQK-LSK108 kits procured from ONT (Oxford Nanopore Technology, UK). Sequencing was performed on GridION X5 (Oxford Nanopore Technologies, Oxford, UK) using the SpotON flow cell (R9.4) in a 48 hr sequencing protocol on MinKNOW 2.1 v18.05.5.

#### Generation of raw data, base-calling, and de-multiplexing

A total of 2,441,632 reads were generated from the pooled library. Nanopore raw reads (*fast5* format) were base called (*fastq* format) and de-multiplexed using Albacore v2.3.1 (ONT, UK).

#### Taxonomic assignment and species identification

The base-called read files were uploaded to the EPI2ME platform via EPI2ME Agent software (ONT, UK). The quality assessment and microbial classification were carried out using the Fastq 16S workflow. Here, reads were first filtered by the quality and then subjected to adapter trimming and barcode detection. Next, taxonomy assignment was carried out using BLAST in conjunction with the NCBI database with a minimum horizontal coverage of 30% and a minimum accuracy of 77% (ONT, UK). Pre-configured alignment parameters such as identity and coverage of sequences were used for analysis.

#### Diversity estimation

The taxonomic assignment results were downloaded as .csv files for each sample for performing downstream analyses such as diversity and taxonomic differential abundance estimation. First, the rarefaction curve analysis was carried out using the modified function ‘ggrare’ (ranacapa R package; Kandlikar et al., 2018) to determine whether sequencing depth was sufficient to discover all the *Pseudomonas* species present in the samples. Next, to identify and remove taxa that are unlikely to be of further use while modelling the data, species having very few counts were filtered out based on their abundance level (minimum counts) across samples (prevalence). Post filtration, data were scaled and normalized for all the samples. This was performed using the MicrobiomeAnalyst software (https://www.microbiomeanalyst.ca/) with default parameters. Further, the community's taxonomic composition and relative abundance were visualized across samples using a stacked bar plot generated by MicrobiomeAnalyst. Here, the top 10 taxa present in each sample were plotted while the ones with very low read counts were merged for better visualization of significant taxonomic patterns.

Diversity estimations were carried out using the ‘phyloseq’ package (R package version 1.19; McMurdie and Holmes, 2013). Alpha-diversity analysis was performed to determine species richness and diversity present within the samples and was calculated based on Chao1 (Chao, 1984), Shannon and Simpson indices (Shannon and Weaver, 1949; Simpson, 1949). Next, to compare the taxonomic diversity across samples, beta-diversity estimations were carried out using the Jenson-Shannon divergence index followed by the Principal Coordinate Analysis (PCoA). These were visualised as a 3D plot, with each point representing the entire microbiome of a single sample.

In addition, *Pseudomonas* species that remain unchanged in their composition across the whole microbial community were identified based on their prevalence and relative abundance in BPH populations using the ‘core’ function in R package ‘microbiome,’ and the heat map was generated using MicrobiomeAnalyst.

Finally, based on the overall similarity and differences in species composition and abundance, hierarchical clustering analysis was performed for BPH populations using the ‘hclust’ function in R package ‘stat’. For this, the Jaccard index was used to measure the distance between samples, and Ward’s linkage algorithm was used for clustering. The result of clustering analysis was represented by a dendrogram generated in MicrobiomeAnalyst.

#### Semi-quantitative PCR (qPCR) for pesticide-exposed and -unexposed BPH

LD_50_/LC_50_ imidacloprid (Confidor 17.80% SL; Bayer AG, Germany) was estimated for the BOD-cultured BPH population to ascertain their pesticide resistance/tolerance status. Further, a pesticide-resistant BPH population was generated in the laboratory by periodically exposing the BOD-cultured BPH population to imidacloprid. Initially, the BOD-cultured BPH population was exposed to LC_40_ imidacloprid solution (0.087%) for the first two generations, but the concentration was gradually increased to 0.162% (LC_60_ imidacloprid) for the subsequent generations. *Pseudomonas* titres in the pesticide-exposed BPH individuals were measured by semi-qPCR. For this, the total genomic DNA was extracted (as mentioned earlier) from individual BPH insects (adults) that were reared under exposure to imidacloprid (1^st^ and 4^th^ generation), and unexposed insects served as the experimental control. The titres of *Pseudomonas* in the pesticide-exposed and -unexposed BPH individuals were compared. The BPH *actin* gene (GenBank: KU196668.1) served as the internal control for normalization. *Pseudomonas*-specific primers Pseudo S2-F and Pseudo S2-R were used to amplify *Pseudomonas* (sequence details as mentioned before), and the primer pair used for *Actin* was ACT-mod F 5’-TGCGTGACATCAAGGAGAAGCTG-3' and ACT-mod R 5’-GTACCACCGGACAGGACAGT-3'. 25 μl of PCR reaction contained 200 μM dNTPs, 0.6 U Taq DNA polymerase (Bangalore Genei, (India) Pvt. Ltd.), 1X Taq buffer and 13 μM of each primer. PCR was performed using 25 ng of the total genomic DNA as a template. The amplification profile used for both *Pseudomonas 16S rRNA* and *Actin* genes was as follows: an initial denaturation of 2 min at 94°C followed by 25 cycles (lesser number of cycles to ensure that subsequent measurements of PCR products were made at the exponential phase of the PCR amplifications) of 30s at 95°C, 30s at 58°C, and 45s at 72°C, with a final extension of 2 min at 72°C. The PCR products were separated on 1% agarose gel and were photographed using a gel documentation system (Alpha Imager, Cell Biosciences, UK). Quantification was done based on the intensity of the band obtained for *Pseudomonas,* and the reaction was normalised using the intensity of the PCR amplified *Actin* gene product from each sample. The relative intensities for each fragment were measured using the Image Lab software 6.0.1 (Bio-Rad Laboratories, USA).

### Quantification and statistical analysis

The statistical significance of the clustering pattern in the 3D plot (generated for beta-diversity estimations) was evaluated using Permutational ANOVA (PERMANOVA; Anderson, 2001) with a p-value cut-off ≤ 0.001. Further, the differentially abundant *Pseudomonas* species across BPH populations were identified using the DESeq2 statistical method for variance estimation (Anders and Huber, 2010) with the adjusted p value cut-off ≤0.05. Additionally, univariate analysis was carried out, and the top 50 species that showed significant variation in abundance between samples were listed. Next, a non-parametric factorial Kruskal-Wallis (KW) sum-rank test (Kruskal and Wallis, 1952) was performed to identify species with significant differential abundance, and this was followed by Linear Discriminant Analysis (LDA) to calculate the effect size of each differentially abundant species (p value cut-off adjusted to 0.05).

## Data Availability

NGS data have been deposited at NCBI as Sequence Read Archive (SRA) files and are publicly available as of the date of publication. Accession number is listed in the key resources table. This paper does not report original code. Any additional information required to reanalyse the data reported in this paper is available from the lead contact upon request.

## Supporting Citations

The following references appear in the Supplemental information: Archana et al. (2020), Frasson et al. (2017), Jha et al. (2009), Kao et al. (2005), Kodama et al. (1985), Lang et al. (2010), Masuda et al. (2007), Romanenko et al. (2005), Simon-Colin et al. (2008), Tao et al. (2014), Thaller et al. (2011), Weon et al. (2006), Xu et al. (2006).

## Supplemental information

Table S1. Mapping statistics of the nanopore sequencing reads obtained for each sample, related to STAR Methods: Taxonomic assignment and species identification

Table S2. Taxonomic classification of the raw reads obtained for each sample upto the genus level, related to STAR Methods: Taxonomic assignment and species identification

Table S3. Data pre-processing, filtering, and normalisation statistics, related to STAR Methods: Taxonomic assignment and species identification

Document S1. Tables S4–S6 and S8

Table S7. Identification of core *Pseudomonas* species as a part of BPH microbiome, related to Figure 8
