# Supplementary material for: Shifts in Pseudomonas species diversity influence adaptation of brown planthopper to changing climates and geographical locations
Source: iScience. 2022 Jun 7;25(7):104550. doi: 10.1016/j.isci.2022.104550 (PMC9218508; doi:10.1016/j.isci.2022.104550)
Supplement: Document S1. Tables S4–S6 — and S8 [file mmc4.pdf]

Table S4 Alpha-diversity indices for estimating the *Pseudomonas* species richness (Chao1) and diversity (Shannon and Simpson) across BPH populations and the TN1 rice samples. Related to Figure 4.

**Samples**	**Chao1**	**Shannon**	**Simpson**
Nalgonda2_1	158	2.83819817	0.89154514
Nalgonda2_2	154.666667	2.62051886	0.85751034
Nalgonda2_3	161.25	2.42900571	0.81218079
August_1	163.333333	2.15081581	0.75826904
August_2	158.1	2.18182845	0.76303264
August_3	158.666667	2.1267294	0.75228152
June_1	154.272727	2.99286518	0.91326502
June_2	143.4	3.17326181	0.93105287
June_3	155.333333	3.14485202	0.92715156
November_1	158	2.59549713	0.78564116
November_2	155.25	1.61413511	0.49222354
November_3	159.333333	2.93609225	0.88575468
DBOD_1	164.333333	1.32171775	0.41055254
DBOD_2	158	2.27549533	0.69162874
DBOD_3	161	2.10499577	0.63869114
Tripura_1	160.333333	2.97699158	0.88488355
Tripura_2	160.375	2.99307874	0.88681018
Tripura_3	160.166667	2.99159792	0.88390984
Nalgonda_1	158.5	2.71446825	0.86817941
Nalgonda_2	159.25	2.76576824	0.87937916
Nalgonda_3	158.75	2.77709344	0.88086108
Manipur_1	160	2.96380335	0.90817178
Manipur_2	160	2.95530358	0.89269648
Manipur_3	158	2.97947549	0.89221769
TN1_1	141.428571	1.80971144	0.65202942
TN1_2	138.47619	3.05087789	0.91713639
TN1_3	142.125	2.55916184	0.84082174

Table S5 Variance estimation and identification of the differentially occurring *Pseudomonas* species across BPH populations as revealed by DESeq2 analysis. Related to Star Methods: Quantification and Statistical Analysis

***Pseudomonas* species**	**log2FC**	**lfcSE**	**Pvalues**	**FDR**
Pseudomonas_knackmussii	3.3399	0.29939	6.72E-29	1.08E-26
Pseudomonas_koreensis	6.6565	0.76381	2.91E-18	2.33E-16
Pseudomonas_fluvialis	-2.2212	0.2579	7.14E-18	3.81E-16
Pseudomonas_glareae	-3.0939	0.40541	2.32E-14	9.29E-13
Pseudomonas_monteilii	-2.885	0.38307	5.03E-14	1.61E-12
Pseudomonas_indoloxydans	-4.1177	0.5579	1.57E-13	4.20E-12
Pseudomonas_azotifigens	4.5256	0.62086	3.12E-13	7.13E-12
Pseudomonas_migulae	-4.4894	0.63141	1.16E-12	2.32E-11
Pseudomonas_oryzae	4.5954	0.66282	4.12E-12	7.32E-11
Pseudomonas_plecoglossicida	-3.2744	0.49285	3.06E-11	4.60E-10
Pseudomonas_otitidis	5.1918	0.78202	3.16E-11	4.60E-10
Pseudomonas_guezennei	5.4015	0.83618	1.05E-10	1.40E-09
Pseudomonas_tolaasii	-4.1665	0.66274	3.24E-10	3.99E-09
Pseudomonas_stutzeri	2.3983	0.39074	8.37E-10	9.56E-09
Pseudomonas_pohangensis	-2.4734	0.43382	1.19E-08	1.24E-07
Pseudomonas_chlororaphis	-2.5704	0.45136	1.24E-08	1.24E-07
Pseudomonas_alcaligenes	2.3416	0.4456	1.48E-07	1.39E-06
Pseudomonas_peli	3.3742	0.65709	2.82E-07	2.51E-06
Pseudomonas_chengduensis	3.3303	0.66526	5.56E-07	4.68E-06
Pseudomonas_viridiflava	-3.5559	0.72902	1.07E-06	8.59E-06
Pseudomonas_soli	3.3257	0.69485	1.70E-06	1.30E-05
Pseudomonas_oryzihabitans	2.9105	0.63036	3.89E-06	2.83E-05
Pseudomonas_balearica	2.1667	0.53054	4.43E-05	0.00030799
Pseudomonas_silesiensis	-2.5327	0.6229	4.78E-05	0.00031892
Pseudomonas_oleovorans	-1.4905	0.36934	5.45E-05	0.00034871
Pseudomonas_zhaodongensis	2.4687	0.61353	5.73E-05	0.00035257
Pseudomonas_seleniipraecipitans	-2.4541	0.61694	6.96E-05	0.00041221
Pseudomonas_fuscovaginae	2.1153	0.54462	0.00010274	0.00058709
Pseudomonas_mendocina	-1.6517	0.42664	0.00010823	0.00059715
Pseudomonas_segetis	-2.2144	0.59008	0.00017499	0.00093329
Pseudomonas_helleri	-2.8406	0.76145	0.00019113	0.00098645
Pseudomonas_hussainii	-4.6743	1.2589	0.00020472	0.0010236
Pseudomonas_borbori	-1.6878	0.46759	0.00030665	0.0014868
Pseudomonas_savastanoi	-3.8697	1.0748	0.00031782	0.0014956
Pseudomonas_toyotomiensis	2.1841	0.61385	0.0003737	0.0017083
Pseudomonas_flavescens	-2.4149	0.68217	0.00040006	0.0017781

Pseudomonas_paralactis	-2.6167	0.74473	0.00044207	0.0019117
Pseudomonas_brenneri	-3.6583	1.056	0.00053149	0.0022378
Pseudomonas_kunmingensis	1.744	0.51595	0.00072413	0.0029708
Pseudomonas_cerasi	-2.7571	0.82681	0.00085392	0.0034157
Pseudomonas_panacis	-4.1921	1.2669	0.00093682	0.0036559
Pseudomonas_guangdongensis	2.227	0.67669	0.00099818	0.0038026
Pseudomonas_nitroreducens	-1.1683	0.3581	0.0011038	0.004107
Pseudomonas_flexibilis	2.017	0.63827	0.001577	0.0057345
Pseudomonas_entomophila	-1.421	0.45689	0.0018692	0.0066459
Pseudomonas_lini	-2.6714	0.90827	0.0032697	0.011273
Pseudomonas_reidholzensis	-1.6871	0.57438	0.0033114	0.011273
Pseudomonas_trivialis	-3.6305	1.269	0.0042239	0.01408
Pseudomonas_weihenstephanensis	2.4367	0.87537	0.0053766	0.017538
Pseudomonas_protegens	-2.6318	0.94959	0.00558	0.017538
Pseudomonas_linyingensis	1.3858	0.5008	0.0056549	0.017538
Pseudomonas_tarimensis	2.5843	0.93481	0.0056999	0.017538
Pseudomonas_aeruginosa	1.2259	0.44498	0.0058715	0.017725
Pseudomonas_coleopterorum	-1.5018	0.56157	0.0074904	0.022194
Pseudomonas_xanthomarina	1.8133	0.6813	0.0077772	0.022625
Pseudomonas_guariconensis	-0.76176	0.29196	0.009076	0.025932
*Pseudomonas_taiwanensis_*	-1.6573	0.64695	0.010417	0.029242
Pseudomonas_sesami	-2.1247	0.84008	0.011433	0.031538
Pseudomonas_donghuensis	1.7232	0.7081	0.014952	0.040548
Pseudomonas_resinovorans	1.4908	0.62031	0.016247	0.043326

*Though DESeq2 variance estimation was performed for all the *Pseudomonas* sp. identified in BPH, only those species which varied significantly between BPH populations (based on their adjusted p-value; cut-off =0.05) are listed here.

FDR: False Discovery Rate; log2FC: log2 Fold Change; lfcSE: standard error of the log2 Fold Change estimate

Table S6 Differentially abundant *Pseudomonas* sp. present across the BPH populations, identified by the Univariate analysis, at the OTU level. Statistical comparisons were performed using the non-parametric Kruskal-Wallis test. (Features were considered to be significant based on their adjusted p-value; cut-off = 0.05). FDR= False Discovery Rate. Related to Star Methods: Quantification and Statistical Analysis

***Pseudomonas* species**	**Pvalues**	**FDR**	**Statistics**
Pseudomonas_benzenivorans	3.42E-11	5.48E-09	56.665
Pseudomonas_kuykendallii	7.86E-11	6.28E-09	51.409
Pseudomonas_oleovorans	2.37E-10	1.26E-08	45.14
Pseudomonas_seleniipraecipitans	6.08E-10	2.43E-08	40.348
Pseudomonas_mendocina	2.33E-09	7.47E-08	34.337

Pseudomonas_hussainii	3.20E-08	8.54E-07	24.911
Pseudomonas_fluvialis	5.94E-08	1.36E-06	23.059
Pseudomonas_indica	1.14E-07	2.27E-06	21.234
Pseudomonas_orientalis	1.28E-07	2.27E-06	20.935
Pseudomonas_sihuiensis	1.73E-07	2.77E-06	20.138
Pseudomonas_pohangensis	2.06E-07	3.00E-06	19.692
Pseudomonas_panipatensis	2.60E-07	3.46E-06	19.119
Pseudomonas_knackmussii	4.33E-07	5.12E-06	17.894
Pseudomonas_glareae	4.48E-07	5.12E-06	17.817
Pseudomonas_azotifigens	5.23E-07	5.58E-06	17.462
Pseudomonas_trivialis	1.09E-06	1.09E-05	15.863
Pseudomonas_oryzae	1.28E-06	1.20E-05	15.523
Pseudomonas_savastanoi	1.40E-06	1.25E-05	15.335
Azomonas_macrocytogenes	2.30E-06	1.94E-05	14.347
Pseudomonas_viridiflava	2.66E-06	2.12E-05	14.075
Pseudomonas_anguilliseptica	2.96E-06	2.22E-05	13.871
Pseudomonas_protegens	3.05E-06	2.22E-05	13.813
Pseudomonas_entomophila	3.49E-06	2.43E-05	13.563
Pseudomonas_nitroreducens	5.61E-06	3.74E-05	12.708
Pseudomonas_citronellolis	7.69E-06	4.92E-05	12.164
Pseudomonas_abietaniphila	8.65E-06	5.32E-05	11.968
Pseudomonas_segetis	2.23E-05	0.0001324	10.46
Pseudomonas_populi	2.33E-05	0.00013298	10.399
Microbulbifer_gwangyangensis	2.43E-05	0.00013421	10.332
Pseudomonas_guariconensis	2.62E-05	0.0001377	10.225
Pseudomonas_flavescens	2.67E-05	0.0001377	10.195
Pseudomonas_straminea	3.66E-05	0.00018294	9.7363
Pseudomonas_borbori	6.02E-05	0.0002921	9.0431
Pseudomonas_delhiensis	6.50E-05	0.0003057	8.9415
Pseudomonas_monteilii	7.42E-05	0.0003394	8.7636
Pseudomonas_aeruginosa	0.00010755	0.00047801	8.2832
Pseudomonas_pelagia	0.00011298	0.00048856	8.2208
Pseudomonas_marginalis	0.0001296	0.00054568	8.0486
Pseudomonas_peli	0.00015111	0.00061993	7.859
Pseudomonas_brenneri	0.00019322	0.00077289	7.5618
Pseudomonas_stutzeri	0.00034304	0.0013387	6.8977
Pseudomonas_taeanensis	0.0005752	0.0021912	6.3334
Pseudomonas_mosselii	0.00063232	0.0023528	6.2333
Pseudomonas_silesiensis	0.00081781	0.0029739	5.9665
Azotobacter_salinestris	0.0010948	0.0038928	5.6724

Pseudomonas_plecoglossicida	0.0014534	0.0050552	5.3952
Microbulbifer_pacificus	0.0020213	0.0067409	5.0826
Pseudomonas_duriflava	0.0020223	0.0067409	5.0821
Azotobacter_beijerinckii	0.0020828	0.0068009	5.0547
Pseudomonas_matsuisoli	0.0022282	0.0071303	4.9922
Pseudomonas_indoloxydans	0.0027536	0.0086388	4.7989
Pseudomonas_thivervalensis	0.0039058	0.012018	4.4887
Pseudomonas_linyingensis	0.0039897	0.012044	4.4702
Pseudomonas_helmanticensis	0.0047114	0.013722	4.3267
Pseudomonas_balearica	0.0047171	0.013722	4.3257
Pseudomonas_xinjiangensis	0.0048357	0.013816	4.3044
Pseudomonas_marincola	0.0050975	0.014309	4.2595
Marinomonas_communis	0.0056002	0.015449	4.18
Pseudomonas_kilonensis	0.0062864	0.017048	4.0833
Methylosarcina_quisquiliarum	0.0070611	0.01877	3.9871
Pseudomonas_lurida	0.0071559	0.01877	3.9761
Pseudomonas_chlororaphis	0.0073364	0.018933	3.9557
Pseudomonas_lutea	0.0076165	0.019344	3.925
Silanimonas_mangrovi	0.0082586	0.020646	3.8591
Pseudomonas_oryzihabitans	0.00873	0.021489	3.8143
Pseudomonas_cremoricolorata	0.010732	0.026016	3.6494
Ventosimonas_gracilis	0.011729	0.02801	3.5794
Pseudomonas_amygdali	0.01203	0.028306	3.5595
Pseudomonas_xanthomarina	0.012281	0.028478	3.5433
Pseudomonas_tolaasii	0.013396	0.030619	3.4758
Pseudomonas_cuatrocienegasensis	0.01381	0.030706	3.4522
Pseudomonas_coleopterorum	0.013818	0.030706	3.4518
Pseudomonas_paralactis	0.014105	0.030717	3.4359
Pseudomonas_composti	0.014207	0.030717	3.4303
Pseudomonas_caspiana	0.015599	0.033278	3.3586
Pseudomonas_umsongensis	0.016274	0.034261	3.3262
Pseudomonas_salina	0.016607	0.034508	3.3108
Pseudomonas_migulae	0.01961	0.040226	3.1853
Pseudomonas_jinjuensis	0.019981	0.040469	3.1713
Pseudomonas_graminis	0.021136	0.042273	3.1294
Pseudomonas_syringae	0.02435	0.0481	3.0246
Pseudomonas_zhaodongensis	0.024714	0.048223	3.0137

Table S8 *Pseudomonas* species showing significant (LDA score >5.8) differential occurrence in BPH populations (as identified by the LEfSe analysis) and their known functional roles. Related to Figure 6.

***Pseudomonas* species**	**Role**	**Reference**
P. mendocina	Detoxification of xenobiotic compounds	Kao et al, 2005
P. glareae	Sugar and protein assimilation, nitrate reduction	Romanenko et al., 2015
P. guezennei	PHA (polyhydroxy alkanoate) producing bacteria	Simon-Colin et al., 2008
P. stutzeri	Pesticide (Parathion) breakdown	Archana et al., 2020
P. chengduensis	Nitrate reduction, heavy metal degradation	Tao et al., 2014
P. indoloxydans	Indole-oxidizing bacterium	Manickam et al., 2008
P. oleovorans	Capable of degrading chloroacetamide herbicide; acetochlor	Xu et al., 2006
P. monteilii	Degradation of aromatic and heterocyclic compounds	Masuda et al., 2007
P. plecoglossicida	Phosphate solubilization, antifungal potential	Jha et al., 2009
P. oryzihabitans	Nitrate reduction (isolated from rice paddies)	Kodama et al., 1985
P. otitidis	Metallo-β-Lactamase production	Thaller et al., 2011
P. reidholzensis	Aesculin hydrolysis, sugar assimilation	Frasson et al., 2017
P. benzenivorans	Xenobiotics degradation	Lang et al., 2010
P. pohangensis	Nitrate reduction, arginine dihydrolase and aesculin hydrolysis	Weon et al., 2006
P. xanthomarina	Denitrifying bacterium	Romanenko et al., 2005
